# Development and Validation of TACE Refractoriness-Related Diagnostic and Prognostic Scores and Characterization of Tumor Microenvironment Infiltration in Hepatocellular Carcinoma

**DOI:** 10.3389/fimmu.2022.869993

**Published:** 2022-04-13

**Authors:** Qifan He, Jian Yang, Yonghai Jin

**Affiliations:** Department of Interventional Radiology, The First Affiliated Hospital of Soochow University, Suzhou, China

**Keywords:** transcatheter arterial chemoembolization (TACE), hepatocellular carcinoma, transarterial embolization refractory, diagnostic score, risk score, immune microenvironment, immunotherapy

## Abstract

**Background:**

Transcatheter arterial chemoembolization LIHC, Liver hepatocellular carcinoma; (TACE) is a valid therapeutic method for hepatocellular carcinoma (HCC). However, many patients respond poorly to TACE, thus leading to an adverse outcome. Therefore, finding new biomarkers for forecasting TACE refractoriness occurrence and prognosis becomes one of the current research priorities in the field of HCC treatment.

**Materials and Methods:**

Based on microarray datasets and a high-throughput sequencing dataset, the TACE refractoriness–related genes (TRGs) were identified by differential expression analysis. LASSO and Cox regression were applied to construct TACE refractoriness diagnostic score (TRD score) and prognostic score (TRP score) and validated their accuracy in external datasets. Functional correlation of TRP score was analyzed by gene set variation analysis and Gene Ontology. CIBERSORT and IMMUNCELL AI algorithms were performed to understand the correlation between the two scores and immune activity. We further carried out the efficacy analysis of immunotherapy and targeted drugs in the different TRP score groups. Furthermore, a nomogram was built by integrating various independent prognostic factors and validated its effectiveness in different datasets.

**Results:**

We identified 487 TRGs combined with GSE104580 and TCGA datasets. Then four novel TRGs (TTK, EPO, SLC7A11, and PON1) were screened out to construct TRD score and TRP score models, and both two scores had good predictive ability in external datasets. Tumors with high TRP score show an immunosuppressive phenotype with more infiltrations of regulatory T cells and macrophages. Immunotherapy and chemotherapy response evaluation revealed patients with a high TRP score demonstrated well reactions to immune checkpoint inhibitors (ICIs) and sorafenib. TRP score, TNM stage, and cancer type were brought into the combined nomogram with optimum prediction.

**Conclusions:**

Our research provided dependable and simplified methods for patients with HCC to assess tumors’ susceptibility to TACE refractoriness and prognosis and guide patients’ clinical therapy choices.

## Introduction

Primary liver cancer is the sixth most common malignant tumor and the fourth most common cause of cancer-related death (1). Hepatocellular carcinoma (HCC) is the most common type of liver cancer. More than 700,000 new cases were diagnosed in the world each year, and half of the new cases were from China (2). The main treatment strategies for HCC are surgery, intervention treatment, radiotherapy, chemotherapy, and palliative therapies; especially, surgery is the best method for early-stage patients (3). Regrettably, 60% to 70% of HCC patients had been intermediate or advanced stage when they were diagnosed, losing the chance for surgical resection (4). More than two-thirds of patients undergoing surgery will recrudesce after 5 years (5). Transcatheter arterial chemoembolization (TACE) is a therapy where drugs aimed to slow or halt tumor development are injected into the artery supplying for HCC tissues (6). According to the Barcelona Clinic Liver Cancer (BCLC) staging system (7), TACE is recommended as the first-line treatment for patients at the intermediate stage (BCLC B) (8). Postoperative overall survival (OS) in HCC patients treated by TACE reaches 70.3% at 1 year, 51.8% at 2 years, 40.4% at 3 years, and 32.4% at 5 years (9).

Anxiously, doctors found that TACE was ineffective for a part of patients, and its efficacy declined as the number of procedures increased in the clinical practice. This phenomenon was first defined as TACE refractoriness/TACE failure by the Japan Society of Hepatology in 2010 (10). Nevertheless, there is not a consentaneous definition of TACE refractoriness/TACE failure in the medical world so far (11). Furthermore, some studies suggest that TACE refractoriness could cause a poor prognosis for patients suffering from HCC (12, 13). Therefore, seeking the risk factors related to TACE refractoriness is critical in liver cancer research. Li et al. considered that the number of tumors and bilobular invasion status were the independent risk factors for TACE refractoriness (14). Niu et al. developed a computed tomography–based radiomics nomogram for the pretreatment prediction of TACE refractoriness (15). Previous studies have noted that some microRNAs, such as microRNA-21, 26a, and 29a-3p, can estimate the probability of TACE refractoriness in HCC patients (16). But diagnostic and prognostic biomarkers of TACE refractoriness have not been intensively studied at present.

Meanwhile, TACE is associated with the regulation of the tumor immune microenvironment. Some research indicates TACE operation can aggravate hypoxic status, promote the generation of proangiogenic cytokines, and induce immunogenic cell death, which gives rise to tumor angiogenesis and changes the tumor immune cell microenvironment (17, 18). Nevertheless, we still lack research on immunological characteristics of TACE refractoriness.

It is now thought that patients need transit from TACE to systemic therapies when TACE refractoriness happens. Sorafenib, a tyrosine kinase inhibitor, has proven statistical significance in multiple clinical trials and is approved for first-line treatment of HCC patients (19, 20). Immune checkpoint inhibitors (ICIs) can regulate and stimulate effective antitumor immune responses, which become a promising strategy in the treatment of cancer (21). Some ICIs for HCC have shown the ability to significantly enhance clinic outcomes (22). Other research indicates the combination of TACE with sorafenib plus ICIs obtains better OS and progression-free survival compared with TACE + sorafenib (23). Regrettably, there is little focused investigation on whether patients with TACE refractoriness are sensitive to ICIs or targeted drugs.

In our study, we focus on the TACE refractoriness–related genes (TRGs) and their diagnostic and prognostic value. TACE refractoriness diagnostic and prognostic scores including four biomarkers (TTK, EPO, SLC7A11, and PON1) performed well in different populations and platforms. Subsequently, we elaborated immune characteristics with two scores and explored their impact on responses to chemotherapy and targeted agents. Furthermore, we built a nomogram model to accurately forecast the prognosis of the patient combined prognostic score and other independent risk factors, benefiting the patient management and treatment in HCC.

## Materials and Methods

### Data Collection

Gene expression data and corresponding prognostic and clinicopathological data of HCC were downloaded from the International Cancer Genome Consortium (ICGC) (24), The Cancer Genome Atlas (TCGA) (25), and Gene Expression Omnibus (26). We obtained GSE104580 cohorts (100 TACE response tissues and 100 TACE nonresponse tissues) and TCGA Liver hepatocellular carcinoma (LIHC) cohorts (374 HCC samples and 50 control samples) for subsequent analyses. The “normalizeBetweenArrays” function in “limma” R package was performed to background adjustment and quantile normalization (27). Besides, a total of 500 HCC samples in GSE14520 and ICGC cohorts, containing gene expression array and prognostic and clinical information, were collected as validation sets. The genes with lower expression and samples with no prognostic information were excluded.

### Differentially Expressed Gene Identification and Functional Annotation

In TCGA and GSE104580, differentially expressed genes (DEGs) between different subgroups were respectively identified utilizing the “limma” package with a fold change of 1.5 and an adjusted *p* < 0.05. Heatmaps for expression of top 50 up-/down-regulated DEGs were drawn by “pheatmap” R package. Venn diagram of the common DEGs between two datasets was visualized by “venndiag” package. We regard these common DEGs as TRGs and further explored their potential functions and enriched pathways using the “clusterprofiler” package in R and *p* < 0.05 was considered as significant (28).

### TACE Refractoriness–Related Prognostic Score

All HCC patients in TCGA were randomly categorized into training (n = 223) and testing (n = 151) sets according to a ratio of 6:4. The TRGs were subjected to univariate Cox regression analysis for identifying those related to OS. Next, the LASSO regression algorithm was conducted to obtain the optimal prognostic genes with 10-fold cross-validation using the “glmnet” R package (29). LASSO regression can improve the accuracy and interpretability of the model and also reduce the risk of overfitting between independent variables (30). Based on these candidate genes, a TACE refractoriness–related prognostic score (TRP score) was established using the multivariate Cox regression. The formula is as follows: TRP score = Σ (Expi * coefi), where Expi represents gene expression level, and coefi denotes risk coefficient. Receiver operating characteristic (ROC) curve analysis was used to illustrate the diagnostic veracity of TRP score in different HCC cohorts (TCGA, ICGC, and GSE14520). A nomogram including candidate genes was constructed using the “survival” and “rms” packages, and its prediction accuracy was evaluated by calibration curves. Patients in the training set were divided into the high-risk group and the low-risk group based on the median TRP score and subsequently carried out to Kaplan–Meier survival analysis. Principal component analysis (PCA) was performed to assess the differences in the distribution of the low- and high-risk groups, and ROC curves were applied to verify the exactness of the score model in TCGA. Simultaneously, to further verify the accuracy of the score model, the testing and external validation sets (ICGC and GSE14520) were also subjected to survival analysis and ROC curve analysis.

### TACE Refractoriness–Related Diagnostic Score

Samples in GSE104580 were randomly divided into training (n = 120) and testing (n = 80) sets. In the training set, identical candidate genes were performed to establish TACE refractoriness diagnostic score (TRD score) using a logical regression algorithm. The relevance between TRP score and TRD score was assessed by Pearson approach. A diagnostic nomogram was constructed to predict the probability of occurrence of TACE refractoriness in HCC patients. Diagnostic ROC curves and calibration plots analyses on account of training sets, test sets, and whole datasets were used to evaluate the efficiency and accuracy of the diagnostic model.

### Clinical Correlation and Stratification Analyses of the TRP Score

To further understand the clinical relevance of the TRP score, we explore the impact of the TRP score on other clinical characteristics (age, sex, Child grade, histologic grade, TNM stage, cancer type, and radiation treatment). The difference in TRP score between clinical subgroups was compared by independent-samples *t* test procedure. In addition, the univariate Cox regression analysis was used to evaluate the correlation between prognosis and clinical characteristics, and multivariate Cox regression analysis was applied to analyze the independent prognostic ability of the risk factors.

### Molecular Mechanism of the Prognostic Signature

Normalized enrichment score of pathways and functional annotation was calculated by the gene set variation analysis (GSVA) method using the “GSVA” R package, and *p* < 0.05 and false discovery rate (FDR) < 0.25 were considered to be statistically significant (31). The pathways significantly distributed between the high- and low-risk groups were selected by *t* tests according to the criteria of *p* < 0.05. The DEGs between two risk stratifications were identified by the “limma” R package (*p* < 0.05, logFC >1.5), and the Gene Ontology (GO) analysis was used to identify the characteristic biological attributes in DEGs. The difference in immune response between two risk subtypes was estimated by gene set enrichment analysis (GSEA) algorithm (32).

### Immune Infiltration Analysis

The ESTIMATE score was calculated using R package “ESTIMATE” to evaluate the composition of the stromal cells and the immune cells (33). CIBERSORT and IMMUNCELL AI algorithms were used to quantify infiltration levels for different immune cell types between high- and low-risk groups using gene expression profiles from the TCGA database (34, 35). The distinction in immune infiltrating level and ESTIMATE score between high- and low-risk groups were analyzed by the Wilcoxon test. To further validate the immune phenotype of different prognostic subtypes, we investigated the distribution of immune molecules, which negatively regulated the antitumor immune response between high- and low-score groups. These immunosuppressive genes were downloaded from the Tracking Tumor Immunophenotype website (http://biocc.hrbmu.edu.cn/) (36, 37).

### Genomics Landscape and Immunotherapeutic Effect

In order to explore the somatic mutations between high- and low-risk groups, the “maftools” R package (37) was applied to analyze the mutation annotation format from TCGA. The somatic mutation data were applied to calculate tumor mutation burden (TMB), an effective immunotherapy predictor (38). Meanwhile, other accepted biomarkers of immunotherapies, such as mismatch repair deficiency (39, 40), HLA genes (41), and immune checkpoint genes (ICPs) (42), were used to reflect the difference in immunotherapeutic sensitivity between high- and low-risk groups. In addition, a series of predicted scores to the checkpoint immunotherapy [cytolytic activity (CYT) score, Tumor Immune Dysfunction and Exclusion (TIDE) score, TME score, and immunophenoscore (IPS)] were conducted to forecast the response to ICP in tumors. CYT score was defined as the geometric mean of PRF1 and GZMA expression (43). Then, we uploaded the HCC transcriptome profiles to the TIDE website (http://tide.dfci.harvard.edu/) and then obtained corresponding TIDE scores (44). TME score, a novel approach to evaluate the efficacy of immune checkpoint blockade, was computed by “TMEscore” R packages (45). The patient’s IPS was obtained from The Cancer Immune Group Atlas (https://tcia.at/home), and a higher IPS is positively correlated to the increased immunogenicity (46). All the aforementioned scores were carried out based on gene expression data from TCGA.

### Drug Susceptibility Analysis

The response to chemotherapy drugs of HCC patients was predicted based on the public pharmacogenomics database Genomics of Drug Sensitivity in Cancer (47). To compare the therapeutic effects of chemotherapeutic drugs in the two different prognostic groups, we measured the semi-inhibitory concentration (IC_50_) values of commonly used chemotherapeutic drugs for LIHC by the “pRRophetic” package (48).

### Establishment and Validation of a Nomogram Scoring System

In accordance with the outcome of the independent prognosis analysis, we developed a predictive nomogram by integrating diverse clinicopathological information, including TNM stage, cancer type, and TRP score, to comprehensively estimate patient survival. Time-dependent concordance index (C-index) and time-dependent ROC analysis was conducted to compare the accuracy of nomogram and different variables by R package “pec” and “riskRegression.” Calibration plots illustrated the consistency between the predicted 1-, 3-, and 5-year endpoint events and the authentic outcomes. Simultaneously, the decision curve analysis of 1, 3, and 5 years was performed to evaluate the clinical utility of the nomogram. Moreover, the external validation of the combined model was carried out based on the ICGC dataset.

### Statistical Analyses

All statistical analyses were conducted *via* R software (version 3.6.7). The Student *t* test was used for statistical comparisons. Spearman correlation was applied for the analysis of the correlation. The Benjamini–Hochberg FDR method was used for *p* value adjustment. Fisher test was used to identify the significant GO terms. *p* < 0.05 was regarded as statistically significant. The cutoff values of continuous variables, such as gene expression and immune infiltration level, were the median.

## Results

### Identification of TACE Refractoriness–Related Genes

**Figure 1:**
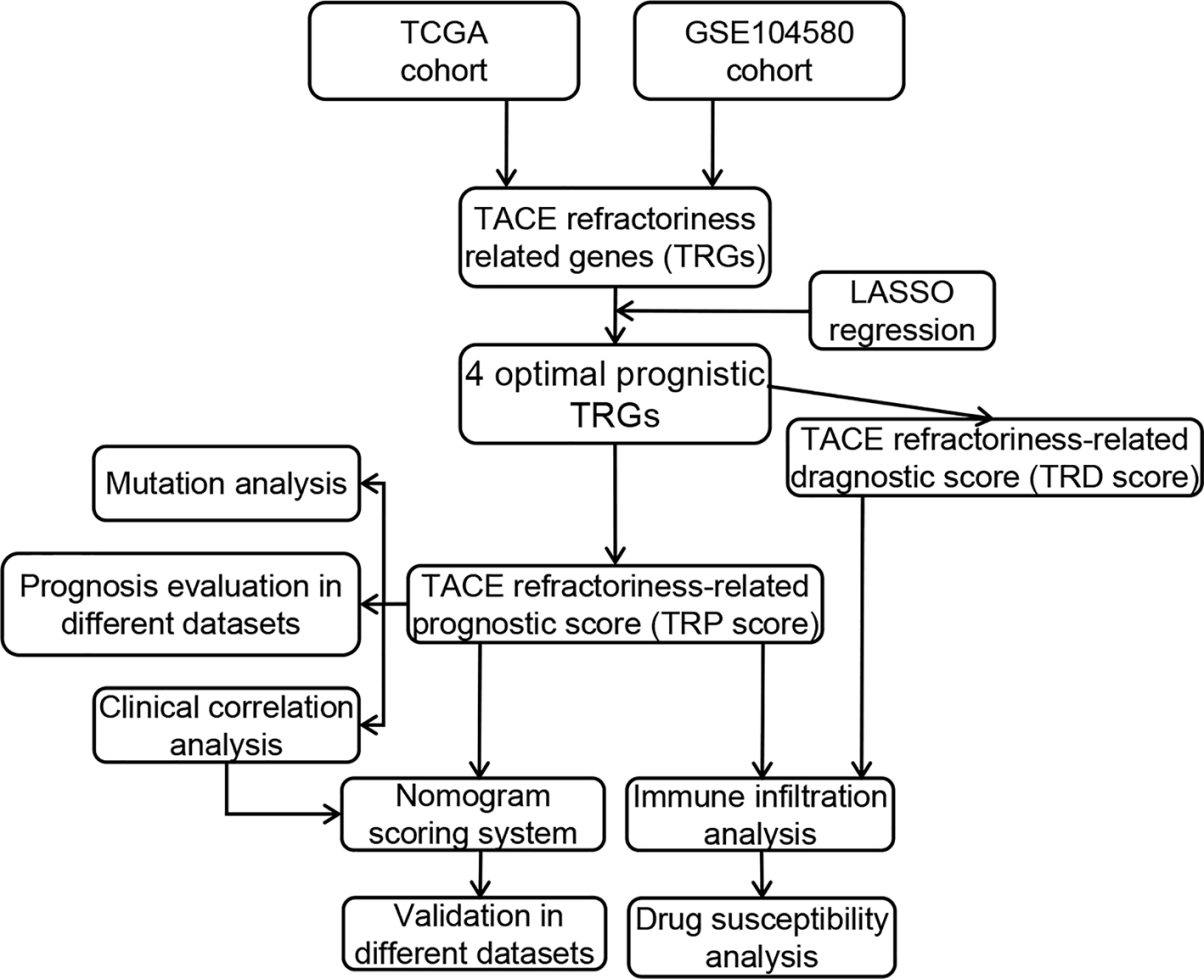
Flowchart of our study.

**Figure 2:**
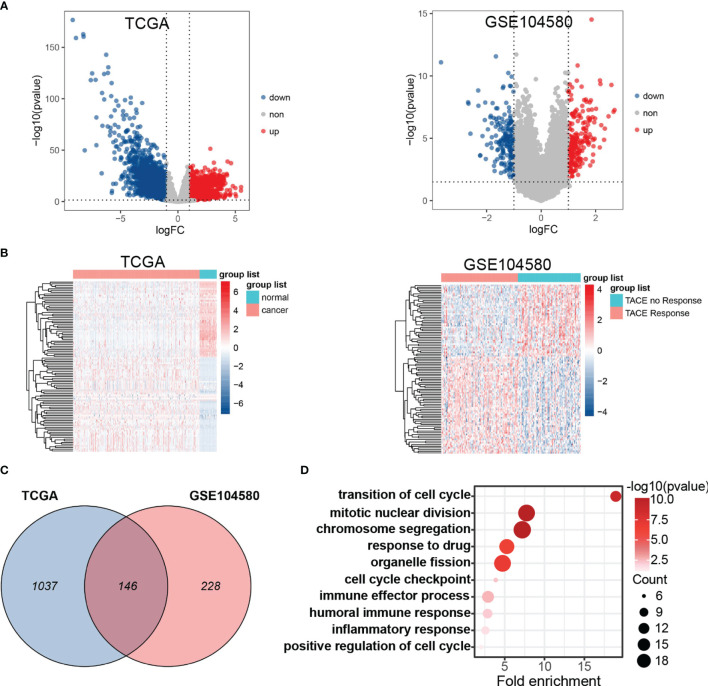
Differential expression gene analysis. **(A)** Volcano plots showed differentially expressed genes (DEGs) in TCGA and GSE104580. **(B)** Hot plot of top 50 up- and down-regulated DEGs in TCGA and GSE104580. **(C)** Venn plot of common DEGs (TRGs) between TCGA and GSE104580. **(D)** Enrichment analysis of TRGs.

The general analysis flow of our study is shown in **Figure 1**. To explore potential TRGs in HCC, we identified 1,183 DEGs from TCGA set and 347 DEGs from GSE104580 set using R package “limma” (**Figure 2A**). Expression patterns of the top 50 up-/down-regulated DEGs of the two datasets are shown in **Figure 2B**. By the intersection of DEGs in two datasets, a total of 487 common DEGs were screened out and named as TRGs (**Figure 2C**). According to the GO annotations, the TRGs were mainly associated with the regulation of cell cycle, chromosome segregation, mitotic nuclear division, regulation of inflammatory response and immune effector process, response to drugs, and organelle fission (**Figure 2D**).

### Construction of the TRP Score Prognostic Model

**Figure 3:**
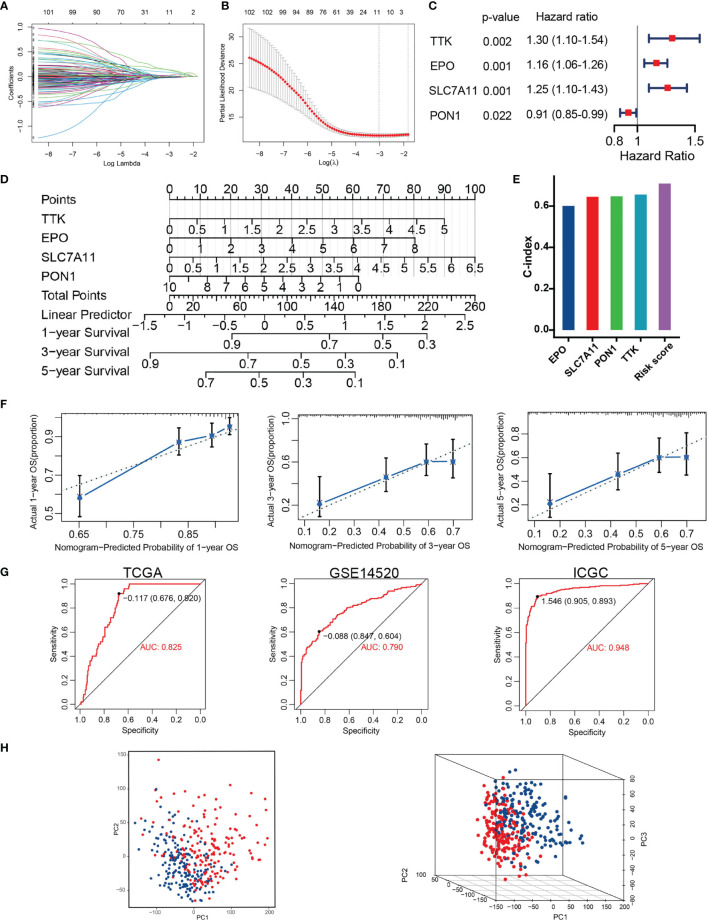
Construction of TACE refractoriness–related prognostic score (TRP score). **(A, B)** LASSO regression analysis of the prognostic TRGs. **(C)** The multivariate Cox regression result. **(D)** Nomogram predicting the probability of patient mortality at 1, 3 or 5 year OS based on four prognostic signatures. **(E)** C-index of various variables. **(F)** Calibration curves of the nomogram. **(G)** ROC curves analysis of the nomogram in TCGA, GSE14520 and ICGC. **(H)** PCA plot showing distribution in the high- and low-risk groups.

Univariate Cox regression analysis found 25 TRGs significantly associated with OS in the training set. Subsequently, four TRGs (TTK, EPO, SLC7A11, and PON1) were further confirmed as the optimal combination parameters of prognosis prediction according to LASSO–Cox regression (**Figures 3A**–**C**). Ultimately, the TRP score was calculated according to the following equation: TRP score = (0.264 ∗ TTK) + (0.147 ∗ EPO) + (0.226 ∗ SLC7A11) + (−0.091 ∗ PON1). We constructed a nomogram incorporating the four markers for calculating TRP score easily and predicting the 1-, 3-, and 5-year OS of tumors roughly (**Figure 3D**). The C-index of TRP score ranked first among all the variables (**Figure 3E**). The calibration curves revealed that the 1-, 3-, and 5-year OS predicted by the nomogram were close to the ideal performance (**Figure 3F**). ROC analysis proved that TRP score exhibited a great diagnostic accuracy for HCC in diverse independent verification datasets, among which area under the ROC curve (AUC) = 0.785 in GSE104580, AUC = 0.825 in TCGA, AUC = 0.825 in TCGA, AUC = 0.79 in GSE14520, and AUC = 0.928 in ICGC (**Figure 3G**). The PCA results demonstrated that patients in the high- and low-risk groups were clearly distributed on both sides (**Figure 3H**).

### Development of the TRD Score Model for Predicting the Possibility of TACE Refractoriness

**Figure 4:**
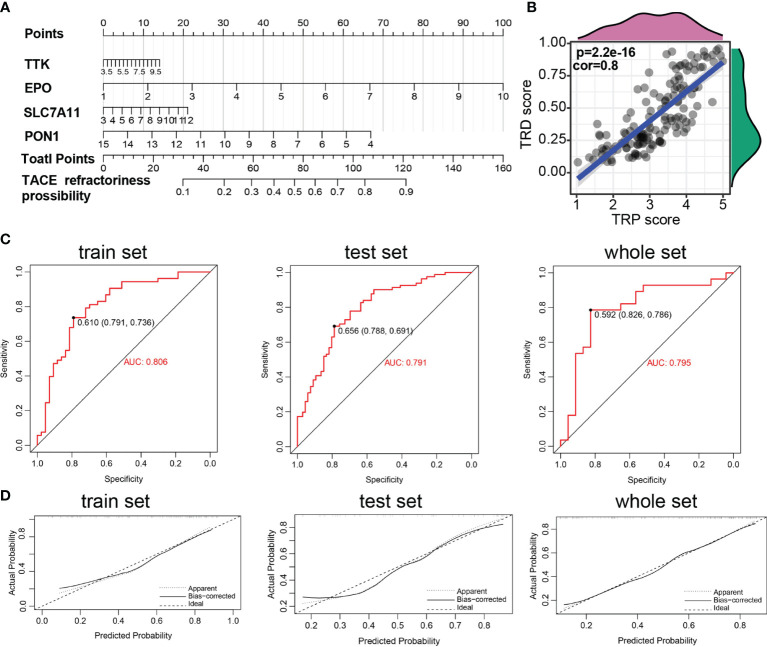
Construction of TACE refractoriness–related diagnostic score (TRD score). **(A)** Nomogram predicting the probability of TACE refractoriness. **(B)** Correlation of TRP score and TRP score. **(C)** ROC curves of the nomogram. **(D)** Calibration curves of the nomogram.

These prognostic signatures were also involved in logical regression to evaluate their diagnostic property on TACE refractoriness based on the training set. The formula of diagnostic score is as follows: TACE refractoriness diagnosis score (TRD score) = (0.315 ∗ TTK) + (0.411 ∗ EPO) + (0.151 ∗ SLC7A11) + (−0.262 ∗ PON1). A diagnostic nomogram for predicting the likelihood of HCC patients suffering TACE refractoriness is shown in **Figure 4A**. There is a strong correlation between the TRP score and TRD score (*p* = 2.2e-16, cor=0.8) (**Figure 4B**), which implies that TACE refractoriness may affect the HCC patients’ outcomes. Diagnostic ROC curves demonstrate that TRD score had excellent prediction performance in training, test, and whole sets (**Figure 4C**). The calibration curves of the diagnostic model presented that predicted values approach actual performance (**Figure 4D**).

### TRP Score Can Effectively Predict Prognosis for HCC Patients

**Figure 5:**
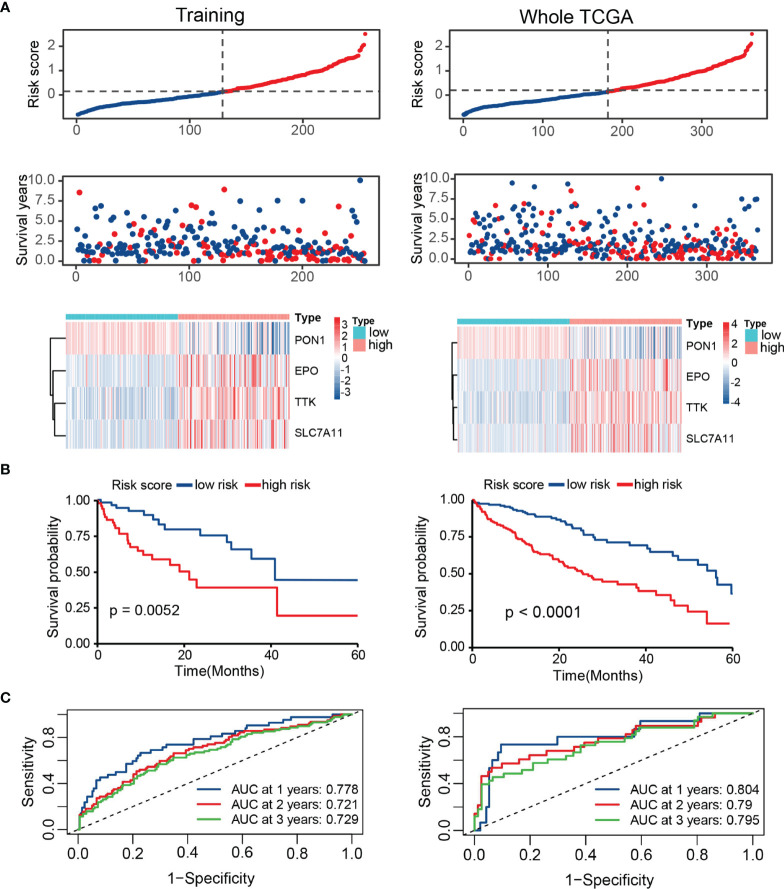
Survival evaluation of TRP score in TCGA. **(A)** Patients ranked by risk score, corresponding survival status and heatmap of the training set and the whole set. **(B)** Kaplan–Meier survival curve of OS in the training set and the whole set. **(C)** ROC curves at 1, 3, and 5 years in the training set and the whole set.

Based on the training set, whole TCGA set, ICGC set, and GSE14520 set, patients were divided into high- and low-risk groups by the median TRP score. The distribution of the risk score and survival status in these datasets was visualized in **Figure 5A** and **S1A**. The expression patterns of four prognostic TRGs were obviously different in the two risk stratifications (**Figure 5A** and **S1A**). Kaplan–Meier survival curves showed a longer OS in patients with low-risk scores than those with high-risk scores (**Figure 5B** and **S1B**). Furthermore, time-dependent ROC curves of risk score displayed high prediction accuracy for OS at 1, 3, and 5 years in several datasets (**Figure 5C** and **S1C**). These results indicate the reliable ability of the TRP score in distinguishing differences in tumors’ outcomes.

### Clinical Correlation Analysis of the TRP Score

**Figure 6:**
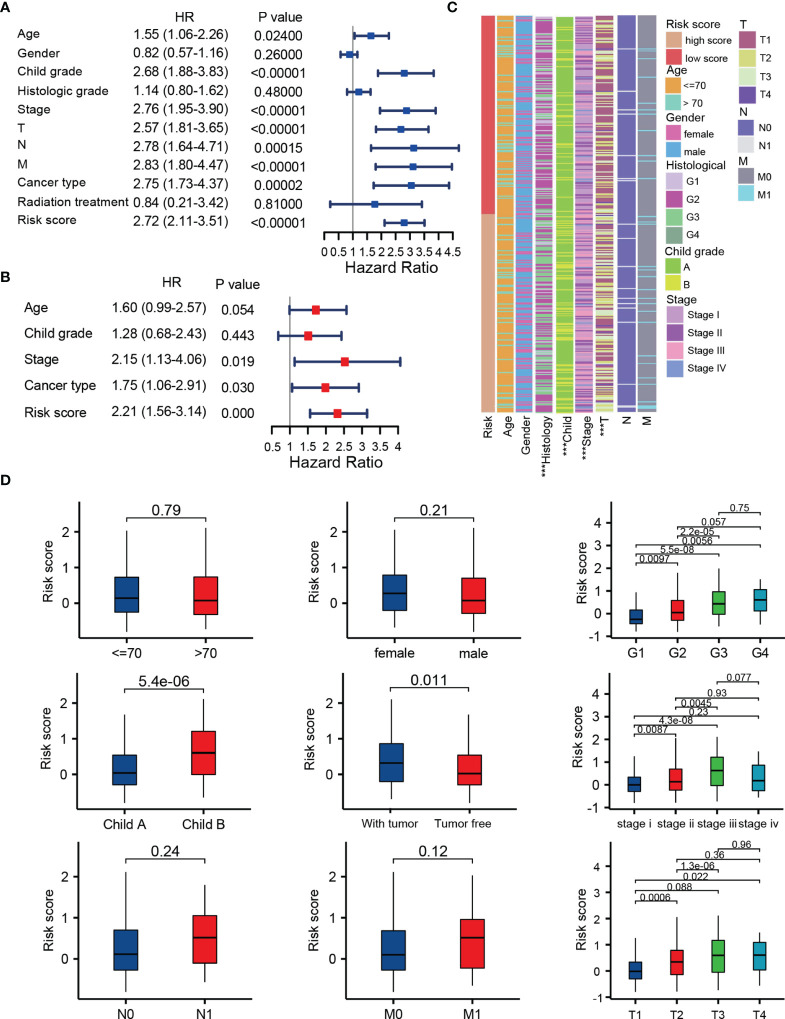
Integration analysis of the TRP score and other clinical factors. **(A, B)** Cox regression analysis of clinical parameters in the whole set. **(C, D)** Correlation of the risk score with different clinical characters ***p < 0.001.

To evaluate the independent prognostic ability of TRP score, we combined a variety of clinical features with the TRP score to perform univariate and multivariate COX regression in TCGA cohorts. As shown in **Figures 6A, B**, TRP score, TNM stage, Child grade, and cancer type showed significant differences, which meant that these indicators could be independent prognostic predictors. Subsequently, we explored the clinical relevance between TRP score and other clinical parameters. The results suggest that TRP score was significantly related to histology grade (*p* < 0.001), Child grade (*p* < 0.001), T stage (*p* < 0.001), and TNM stage (*p* < 0.001), and it gradually elevated with the development of these clinical factors (**Figures 6C, D**).

### The Potential Molecular Mechanism of the Prognostic Signature

**Figure 7:**
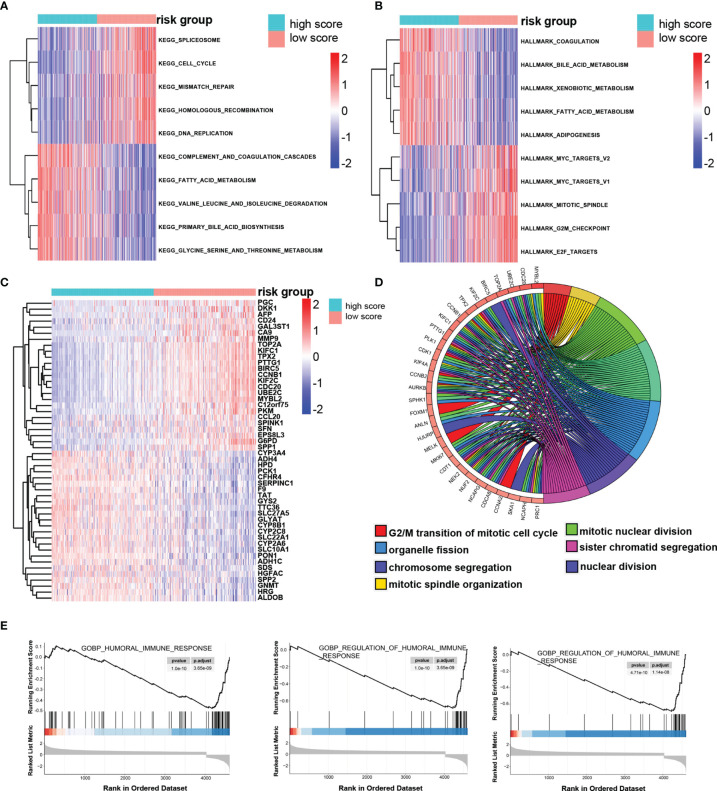
The potential molecular mechanism of the TRP score. **(A, B)** GSVA-KEGG and GSVA-HALLMARK for the TRP score. **(C)** The heatmap of DEGs between high- and low-TRP score groups. **(D)** GO function annotation of DEGs. **(E)** GSEA using immune gene set.

Through GSVA algorithm to estimate the variation of pathway activity in the TCGA sample population, we found that the high-risk group was significantly enriched for the cell cycle, such as homologous recombination, mitotic spindle and G2/M checkpoint, and those related to the DNA repair, such as mismatch repair, whereas the activity of pathways related to metabolism, such as fatty acid metabolism, glycine, serine, and threonine metabolism, enriched significantly in the low-score group (**Figures 7A, B**). The up-regulated DEGs in the high-risk group significantly enriched in those terms related to mitotic nuclear division, chromosome segregation, mitotic spindle organization, and nuclear division, which is in keeping with the result of GSVA and implied that the molecular signature of the high-score group was closely associated with the cell cycle (**Figures 7C, D**). It is worth noting that the immune responses were more active in the low-risk group rather than the high-risk group, as revealed by GSEA (**Figure 7E**).

### Immune Landscape in the Low- and High-Risk Group

**Figure 8:**
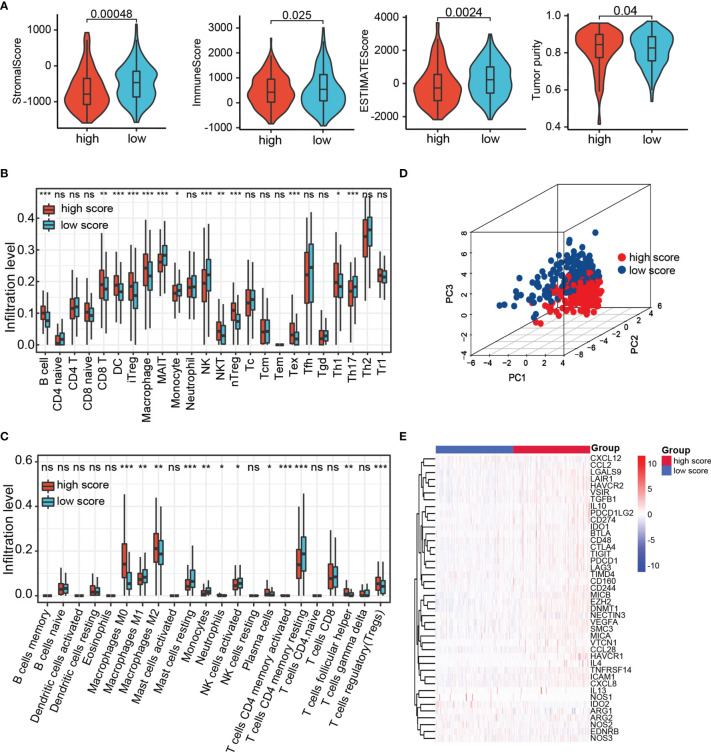
The landscape of tumor immune microenvironment. **(A)** StromalScore, ImmuneScore, ESTIMATEScore, and Tumor purity between high- and low-risk group in TCGA. **(B, C)** Relative cell abundance immune cells by CIBERSORT and IMMUNECELL AI between two groups in TCGA. **(D)** PCA performed on HCC patients based on significant differences in immune cells between high- and low-risk groups. **(E)** Differentially expressed genes involved in the negative regulation of the cancer–immunity cycle between high- and low-risk groups. ns *p* > 0.05, *p < 0.05, **p < 0.01, ***p < 0.001.

The infiltration of immune cells in HCC was estimated by three independent methods (CIBERSORT, IMMUNECELL AI, and ESTIMATE) to evaluate the interaction of prognostic score with tumor immune microenvironment. The immune score, stromal score, and ESTIMATE score of the high-risk group were lower than those in the low-risk group, whereas tumor purity was higher in the high-risk group (**Figure 8A**). After examining the results of CIBERSORT and IMMUNECELL AI, we found that regulatory T cells (Tregs) and macrophages were both significantly elevated in high-risk group (**Figures 8B, C**). In addition, based on 22 immune cell subpopulations from CIBERSORT, patients with high- and low-risk could be clearly divided into two discrete groups by PCA analysis (**Figure 8D**). As shown in **Figure 8E**, the immunosuppressive genes were essentially up-regulated in high-risk group. On the other hand, with the help of CIBERSORT and IMMUNECELL AI algorithms, we found that macrophages increased in the TACE nonresponse group (**Figures S2A, B**). This evidence indicates that patients with high TRP score and TACE refractoriness had low activities of antitumor immune processes.

### TRP Score Was a Predictive Biomarker for Clinical Response to HCC Immunotherapy

**Figure 9:**
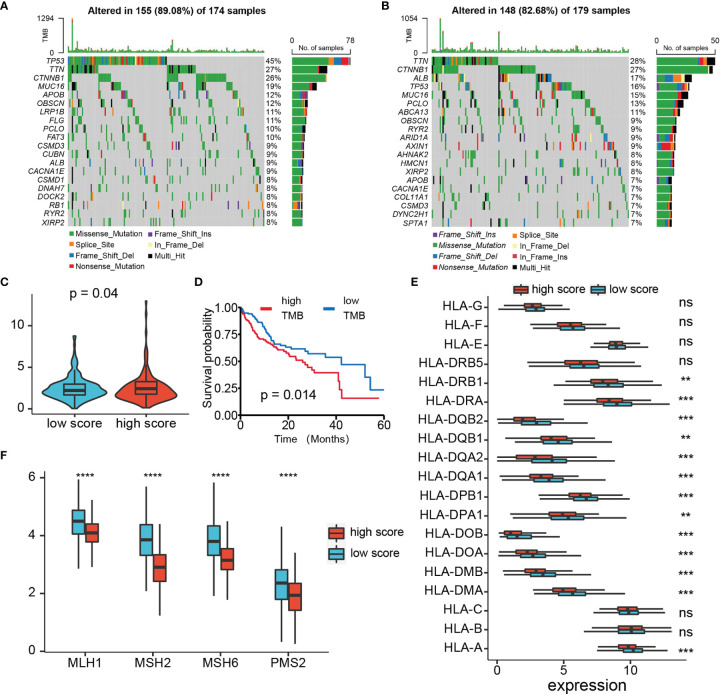
Analysis of TMB, MMR, and HLA expression. **(A, B)** Top 15 gene mutations in high- and low-risk tumors. **(C)** Box plot of TMB of HCC patients between high- and low-risk groups. **(D)** Kaplan–Meier plot of overall survival for patients in high- and low-TMB groups. **(E)** The profile of HLA genes expression levels in high- and low-risk groups. **(F)** The profile of MMR-related genes expression levels in high- and low-risk groups. ns *p* > 0.05, **p < 0.01, ***p < 0.001, ****p < 0.0001.

**Figure 10:**
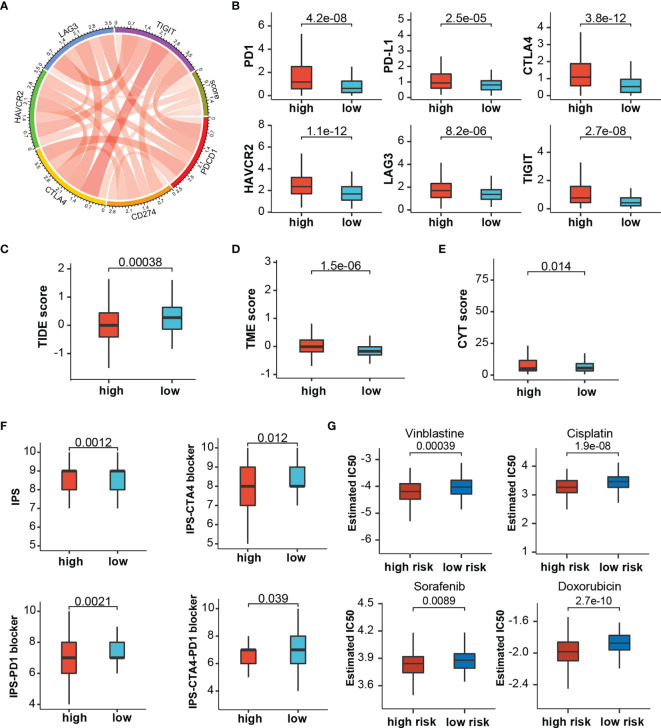
The estimation of Immunotherapy and chemotherapy response. **(A)** Circos plot showing the correlation between immune checkpoints and the risk score. **(B)** The expression levels of immune checkpoints between risk stratifications. **(C–F)** The association between risk score and TIDE score, TME score, CYT score, and IPS. **(G)** The chemotherapy response of two prognostic subtypes for four common chemotherapy drugs.

Through the mutation landscape of HCC patients, we discovered a higher TP53 mutation rate existed in the high-score group rate than the low-score group (45% vs. 16%, **Figures 9A, B**). The TMB slightly increased in the high-score group (**Figures 9C**), and high TMB suggested a bad prognosis (**Figures 9D**). Meanwhile, preferential expression of MMR-related genes (MLH1, MSH2, MSH6, and PMS2) and HLA genes were found in the low-risk group (**Figures 9E, F**). Six immune checkpoint molecules (CTLA4, PD1, PD-L1, LAG3, HAVCR2, and TIGIT) were significantly up-regulated in the high-risk group (**Figures 10A, B**). To evaluate the impacts of TRP score on the tumors’ response to ICP therapy, we analyzed the correlation between TRP score and multiple immunotherapeutic indices. The results demonstrated that the TRP score was actively associated with the CYT score and TME score, while negatively associated with the TIDE score (**Figures 10C**–**E**). Furthermore, IPS analysis indicated the IPS, IPS-CTLA4, IPS-PD1, and IPS-PD1-CTLA4 scores were higher in the high-score group (**Figure 10F**). As for TACE refractoriness, higher ICP (PD1, HAVCR2, and TIGIT) expression and lower TIDE score mainly concentrated in the TACE nonresponse group (**Figure S2C**). Thus, these results revealed that TRP score may serve as targets for immunotherapy, and patients with high-TRP and TACE refractoriness seem more sensitive to ICPs.

### High-Risk Group Were Sensitive to Multiple Chemotherapeutic Drugs

Based on TCGA cohorts, we calculated the IC_50_ of common chemotherapeutic drugs recommended for liver cancer treatment by the American Joint Committee on Cancer guidelines to evaluate the sensitivities of patients in the low- and high-risk groups to these drugs. We discover that the patients in the high TRP score group had lower IC_50_ value for sorafenib, doxorubicin, vinblastine, and cisplatin (**Figure 10G**). Meanwhile, patients with TACE refractoriness showed lower IC_50_ value for sorafenib (**Figure S2D**). These results indicated that TRP score and TACE refractoriness can significantly impact patients’ sensitivity to some drugs.

### Development and Verification of a Predicted Nomogram Integrated TRP Score and Other Clinical Characters

**Figure 11:**
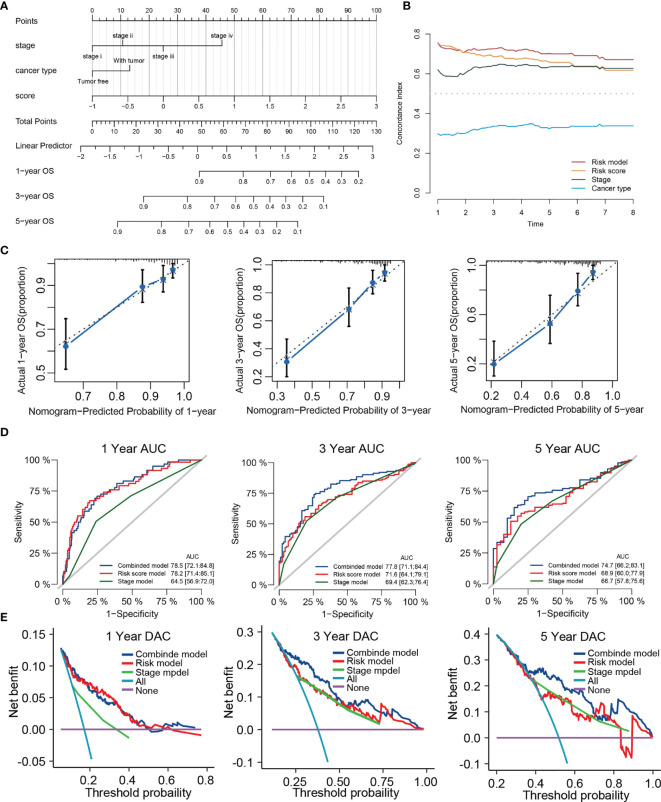
Construction of a nomogram predicting OS based on the independent prognostic factors. **(A)** Nomogram integrated the TRP score, Stage and cancer type. **(B)** Time-dependent C-index curves of nomogram, TRP score cancer type and stage. **(C)** Calibration curve for predicting OS at 1, 3, and 5 years. **(D)** Time-dependent ROC curves of nomogram, TRP score, and stage at 1, 3, and 5 years. **(E)** Decision curve analysis of nomogram, TRP score and stage at 1, 3, and 5 years.

For synergizing clinical efficacy of PRG score in the prognosis, we established a nomogram containing the PRG score and other clinicopathological characteristics to predict the 1-, 3-, and 5-year OS with better precision (**Figure 11A**). Time-dependent C-index analysis demonstrated that the nomogram exhibited superior predictive performance about survival rate compared with other clinical parameters (**Figure 11B**). The calibration curves of the nomogram of 1-, 3-, and 5-year OS prediction were approached to the ideal model (**Figure 11C**). Meanwhile, time-dependent ROC curves at 1-, 3-, and 5-year OS indicated that the nomogram possessed the best predicting precision compared with TRP score and TNM stage (**Figure 11D**). DCA at 1-, 3-, and 5-year OS proved the nomogram had great clinical utility (**Figure 11E**). As for validation analyses in the external dataset (ICGC-LIHC), the nomogram showed the same prognostic value through comprehensive evaluation with time-dependent C-index, calibration curve, and decision curve (**Figure S3**).

## Discussion

HCC is a common digestive system tumor with high aggressiveness and poor prognosis. HCC is insensitive to conventional radiotherapy and chemotherapies; consequently, surgery becomes the main treatment (49). Besides, only 30% to 40% of LIHC patients are eligible for surgical resection, and the recurrence rate after surgery is very high (50). Hence, TACE is one of the current standard treatments for unresectable HCC patients, especially those with BCLC stage B (intermediate stage). Recently, TACE refractoriness has become a thorny issue and received more and more attention, whose definition is tumor progression after TACE treatment. Moreover, Post-TACE will cause complex immune regulation, and the exposure of damage-associated molecular patterns can activate antigen processing cells, which have phagocytosed tumor antigens (51).

A series of research identify some predictive factors or models related to TACE refractoriness. Granito et al. found posttreatment transient transaminase elevation was predictive of objective response to superselective cTACE. But most other studies focus on radiological features or common tumor markers rather than specific biomarkers (52). Second, few studies focus on the relation between TACE refractoriness and HCC patient outcome, and developed predictors are primarily used for diagnosis rather than prognosis. So far, no studies have explored the correlation between TACE refractoriness and immune microenvironment. Therefore, the emphasis of this study lies in constructing diagnostic and prognostic models that have well prediction performance on preoperative risk of TACE refractoriness and survival possibility and try to explain their potential immune mechanism.

In our research, we first identify 487 common TRGs between the TCGA cohort and GSE104580 cohort. Through Cox regression and LASSO algorithm, four TRGs were identified, which are significantly associated with the prognosis of LIHC and further constructed diagnostic and prognostic score models. Among them, TTK, EPO, and SLC7A11 are the risk factors, and PON1 is a protection factor for the prediction of TACE refractoriness and outcome. TTK is a dual-specificity protein kinase that abnormally expresses in various cancers, including breast cancer and prostate cancer. Studies have indicated that TTK was actively associated with acquired sorafenib refractoriness and can be a therapeutic target in HCC (53, 54). EPO encoded erythropoietin to promote red blood cell production. Wei et al. have reported that high EPO in blood before TACE is noticeably related to poor response to TACE (55). SLC7A11, a critical regulator of ferroptosis, overexpresses in multiple human cancers to promote tumor growth through suppressing ferroptosis (56). Lyu et al. reported that the circ0097009/miR-1261/SLC7A11 axis is involved in the progression of HCC (57). PON1 can act as a biomarker for the estimation of microvascular invasion and evaluation of prognosis in HCC (58). Both diagnostic and prognostic scores exhibit good predictive performance and have a significant positive correlation. It is understandable that tumors with TACE refractoriness lose the good opportunity of palliative treatment and will lead to an adverse outcome. The ROC analysis confirmed the great utility of TRP score for predicting the 1-, 3-, and 5-year survival rates in different HCC cohorts. Clinical correlation analysis presented that TRP score was significantly different in different clinical subgroups, including clinical stage, Child grade, histology grade, and T stage. In addition, multivariate Cox regression analysis verified that TRP score could serve as an independent prognostic signature. We further developed a nomogram prognostic model containing TRP score, stage, and cancer type. Interestingly, we discovered the combined model had the highest AUC and net benefit compared with other single factors for predicting survival, which were corroborated in TCGA and ICGC cohorts. These results proved that the integrated nomogram was optimum prognostic model compared with other clinical factors.

Through the research on the molecular mechanism of prognostic signatures, we found these TRGs were also characterized by cell division and immune regulation, such as regulation of cell cycle, chromosome segregation, mitotic nuclear division, regulation of inflammatory response, and immune effector process. This implies that TRGs may be involved in tumor progression and immune microenvironment. With the help of GSVA and GSEA, we found that the high-score group with a poor prognosis was remarkably enriched in the active metabolism, whereas the high-score group with a poor prognosis not only exhibited low immune response and metabolic activity but also involved cell cycle regulation. An active metabolism was considered as one of the important signatures of a good prognosis of HCC (59).

As the continuous breakthrough in the field of cancer immunotherapy, more and more research supports that the tumor microenvironment can regulate cancer progression (60). To evaluate the relationship between TRP score and immune infiltration, we used the CIBERSORT and IMMUNECELL AI to analyze immune cell proportions in two score groups. The results showed that Tregs and macrophages were significantly higher in the high-risk group. CD4^+^CD25^+^Foxp3^+^ Tregs are the immunosuppressive cells related to the tumor progression, invasiveness, as well as metastasis. It is worth noting that Tregs can represent a direct target of ICI immunotherapy because several checkpoint molecules including CTLA4 and PD-1 are highly expressed on Tregs. Hence, the targeting of Tregs by ICI immunotherapy may lead to the development of immune-related adverse events (irAEs), such as severe T cell–mediated autoimmune disease (61). Tumor-associated macrophages (TAMs) are shaped by chemokines such as IL-4, IL-10, and IL-13 to suppress antitumor immunity (62). Meanwhile, we further found that most immunosuppressive molecules positively expressed in high-risk group. Thus, we believe that high-risk tumors exhibited a more suppressive immune phenotype due to higher cell abundance of infiltrate Tregs and TAMs. In addition, TAMs were also higher in the high-TRD score group, which indirectly validated that the immunosuppressive phenotype existed in patients with TACE refractoriness.

Compared with current treatment options such as surgery, chemoradiotherapy, liver transplantation, and radiofrequency ablation, immunotherapy is a novel and promising treatment approach against HCC (63). High levels of ICP, TMB, and HLA genes, as well as low levels of MMR genes, are usually considered to be predictors for tumors suitable for immunotherapies. TMB is a good indicator to assess the neoantigen load and predict the ICI response. Defective DNA mismatch repair causes abundant mutant neoantigens in cancer, and low expression of MMR genes may lead to defective MMR and enhance the cancer sensitivity to ICI (64). Simultaneously, a decrease in HLA expression may weaken cells’ ability to present neoantigens and induce immune evasion (41). ICIs such as PD-1 and PD-L1 provided a major breakthrough for tumor treatment, and clinical evidence has demonstrated their efficacy and safety. Our study revealed multiple immune checkpoints (like PD-1, PD-L1, and CTLA4), HLA genes, and TMB up-regulated, whereas TMB reduced in the high-score groups. Furthermore, various prediction scores such as CYT score, TIDE score, TME score, and IPS were widely used to predict tumors’ response to ICIs. Higher CYT score, TME score, and IPS mean more effective response to ICIs therapy, but TIDE score is completely opposite. Strikingly, we observed that TME scores, CYT score and IPS significantly enhanced, whereas TIDE score diminished in high-risk groups compared with low-risk groups. In addition, TRD score was significantly associated with ICI expression and TIDE score, which demonstrated that immunotherapy was suitable for cases with TACE refractoriness. Therefore, the above results demonstrate that tumors with high TRP scores might be more sensitive to ICIs and indirectly suggested for patients with TACE refractoriness that immunotherapy could be an effective solution.

It is no doubt that the development of locoregional treatment in HCC potentially improves the survival of HCC patients, such as liver translation, radiofrequency ablation, percutaneous ethanol injection ablation, and TACE (65). Sorafenib is the first-line treatment for HCC, and it can inhibit tumor angiogenesis by targeting the RAF-MEK-ERK signaling pathway or blocking the expression of vascular endothelial growth factor receptor (66). Drug susceptibility analysis found that the IC_50_ value for sorafenib was lower in the high-risk group, which meant patients with high TRP scores might have a better response toward sorafenib treatment. From recent materials, sorafenib + ICI combination treatment can prolong survival time in those patients with TACE refractoriness and advanced HCC (23), which in keeping with our findings that sorafenib seems more effective for TACE refractoriness patients.

Nonetheless, several limitations were notable in our study. First, as all data were collected retrospectively from public databases, the potential bias of clinicopathological features is inevitable. Thus, large-scale prospective studies and functional and mechanistic experimental studies are needed to support our findings. Second, there is still a debate in the medical community about a clear definition of TACE refractoriness. Only one TACE refractoriness dataset was included in the study, and it lacks complete clinical information. So, we need more studies to verify and improve the predictive power of the diagnostic score. Finally, we selected only patients with RNA-seq data to analyze immunotherapy response in HCC; this selection bias might cause the prediction error for signatures.

In conclusion, our study developed a novel prognostic score and a diagnostic score related to TACE refractoriness for HCC. The two signatures reveal the close relationship between TACE refractoriness and immunosuppression in HCC. They also exhibit a strong ability in predicting response to ICIs. Moreover, a nomogram developed based on the prognostic score with a strong capacity of predicting HCC outcomes deserves promotion in clinical practice.

## Data Availability Statement

The datasets analyzed was acquired from The Cancer Genome Atlas (TCGA) database (https://portal.gdc.cancer.gov/) and ICGC database (https://dcc.icgc.org/).

## Author Contributions

QH designed the experiments and data collection and wrote the paper. JY and YJ were responsible for manuscript review and providing constructive comments. All authors approved the final manuscript.

## Conflict of Interest

The authors declare that the research was conducted in the absence of any commercial or financial relationships that could be construed as a potential conflict of interest.

## Publisher’s Note

All claims expressed in this article are solely those of the authors and do not necessarily represent those of their affiliated organizations, or those of the publisher, the editors and the reviewers. Any product that may be evaluated in this article, or claim that may be made by its manufacturer, is not guaranteed or endorsed by the publisher.

## Supplementary Material

Supplementary Figure 1 Survival evaluation of TRP score in ICGC and GSE14520. **(A)** Patients ranked by risk score, corresponding survival status and gene expression heatmap of ICGC and GSE14520. **(B)** Kaplan–Meier survival curve of OS in the ICGC and GSE14520. **(C)** ROC curves at 1, 3, and 5 years in the ICGC and GSE14520.

Supplementary Figure 2 Immune infiltration and drug sensitivity analyses based on two TACE refractoriness stratifications. **(A, B)** Relative cell abundance immune cells by CIBERSORT and IMMUNECELL AI between TACE nonresponse and TACE response groups in GSE104580. **(C)** The correlation of TACE refractoriness with immune checkpoints and TIDE score. **(D)** The chemotherapy response of two TACE refractoriness subtypes for three common chemotherapy drugs.

Supplementary Figure 3 Validation analysis of nomogram in ICGC. **(A)** Time-dependent ROC curves of nomogram at 1, 3, and 5 years. **(B)** Time-dependent C-index curves of nomogram. **(C)** Decision curve analysis curves of nomogram at 1, 3, and 5 years. **(D)** Decision curve analysis of nomogram, TRP score and stage at 1, 3, and 5 years.

## Abbreviations

HCC, Hepatocellular carcinoma; LIHC, Liver hepatocellular carcinoma; TACE, Transcatheter arterial chemoembolization; TCGA, The Cancer Genome Atlas; ICGC, International Cancer Genome Consortium; TRGs, TACE refractoriness–related genes; TRD score, TACE refractoriness prognostic score; TRD score, TACE refractoriness diagnostic score; GSEA, Gene set enrichment analysis; GO, Gene Ontology; KEGG, Kyoto Encyclopedia of Genes and Genomes; ROC, Receiver operating characteristic; K-M, Kaplan–Meier; AUC, Area under the ROC curve; PCA, Principal component analysis; TNM, Tumor size/lymph nodes/distance metastasis; ICIs, Immune checkpoint inhibitors.
