# Supplementary figures and images for: Development and Validation of TACE Refractoriness-Related Diagnostic and Prognostic Scores and Characterization of Tumor Microenvironment Infiltration in Hepatocellular Carcinoma
Source: Front Immunol. 2022 Apr 13;13:869993. doi: 10.3389/fimmu.2022.869993 (PMC9043752; doi:10.3389/fimmu.2022.869993)

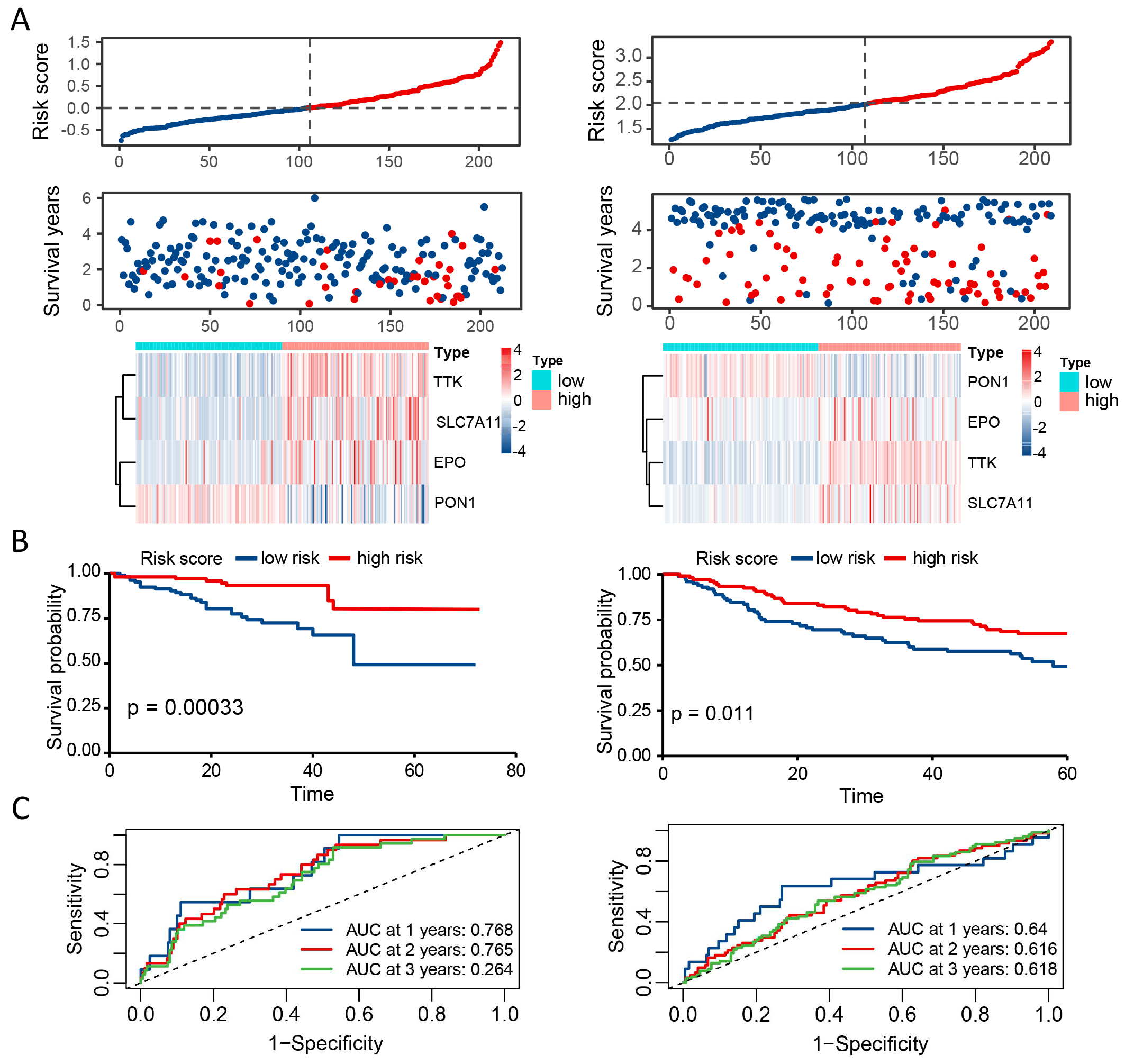

Supplement: Supplementary Figure 1 — Survival evaluation of TRP score in ICGC and GSE14520. (A) Patients ranked by risk score, corresponding survival status and gene expression heatmap of ICGC and GSE14520. (B) Kaplan–Meier survival curve of OS in the ICGC and GSE14520. (C) ROC curves at 1, 3, and 5 years in the ICGC and GSE14520. [file Image_1.tif]

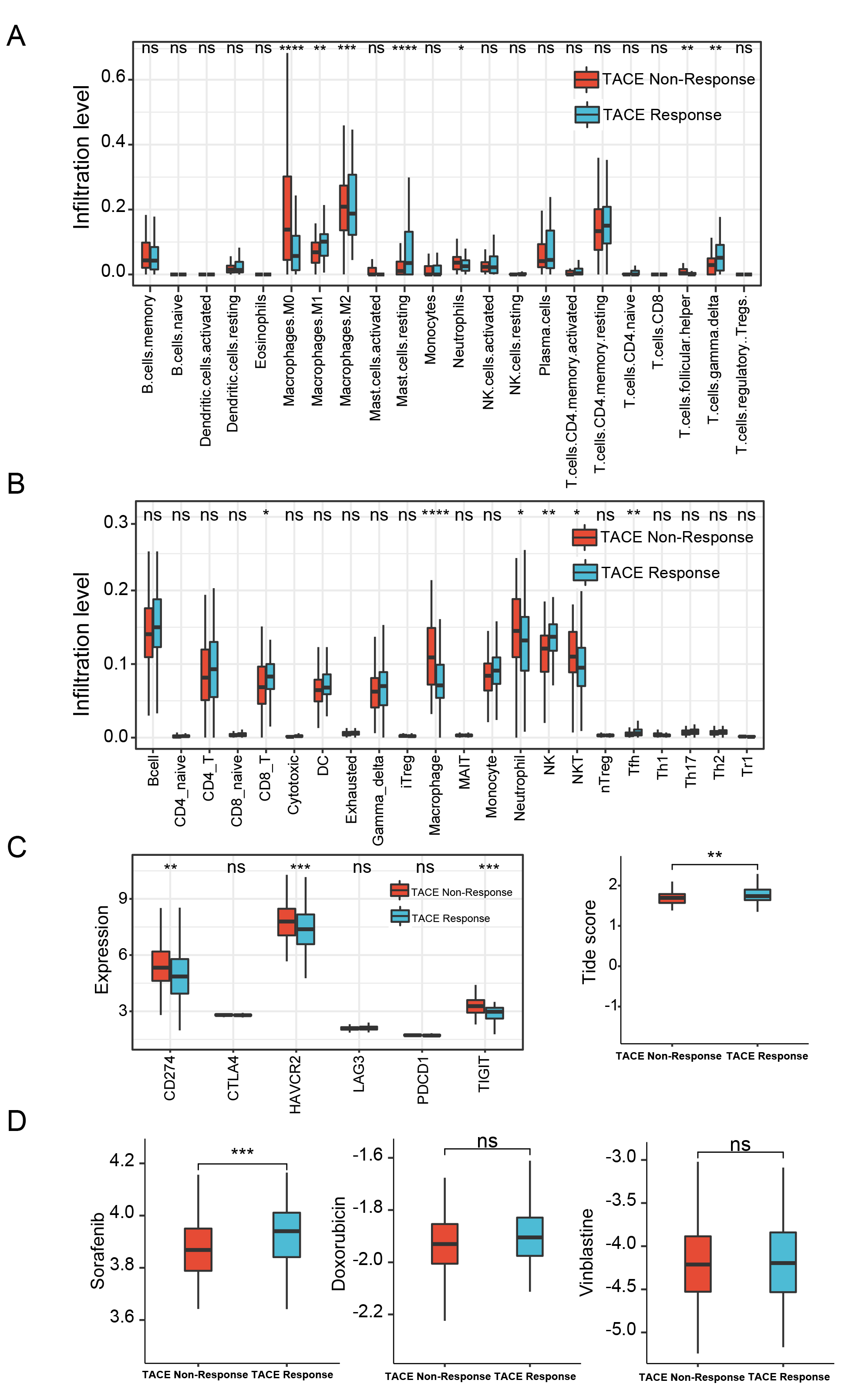

Supplement: Supplementary Figure 2 — Immune infiltration and drug sensitivity analyses based on two TACE refractoriness stratifications. (A, B) Relative cell abundance immune cells by CIBERSORT and IMMUNECELL AI between TACE nonresponse and TACE response groups in GSE104580. (C) The correlation of TACE refractoriness with immune checkpoints and TIDE score. (D) The chemotherapy response of two TACE refractoriness subtypes for three common chemotherapy drugs. [file Image_2.tif]

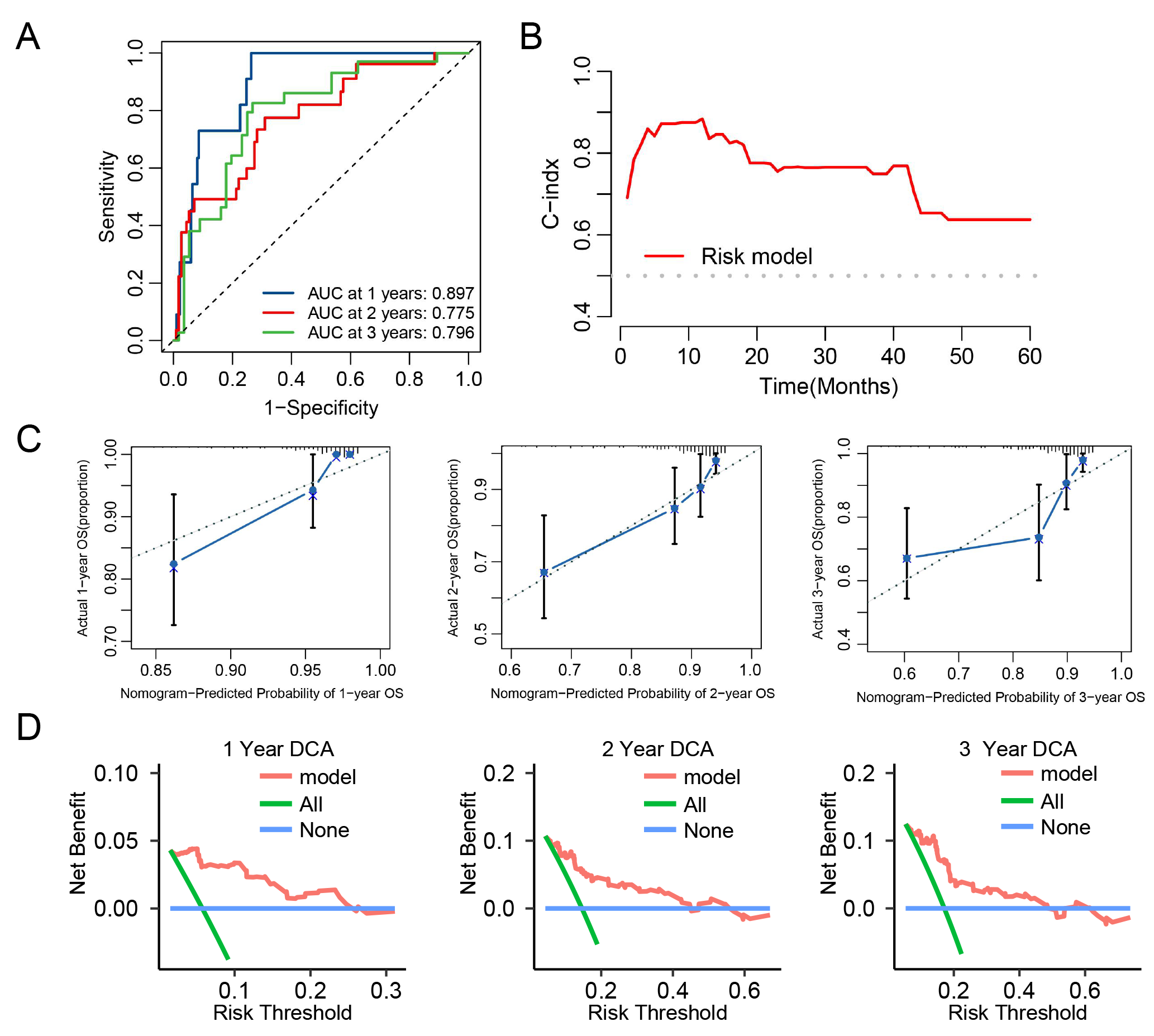

Supplement: Supplementary Figure 3 — Validation analysis of nomogram in ICGC. (A) Time-dependent ROC curves of nomogram at 1, 3, and 5 years. (B) Time-dependent C-index curves of nomogram. (C) Decision curve analysis curves of nomogram at 1, 3, and 5 years. (D) Decision curve analysis of nomogram, TRP score and stage at 1, 3, and 5 years. [file Image_3.tif]
